# Body fat anthropometric indexes: Which of those identify better high cardiovascular risk subjects? A comparative study in Spanish population

**DOI:** 10.1371/journal.pone.0216877

**Published:** 2019-05-23

**Authors:** Arturo Corbatón Anchuelo, María Teresa Martínez-Larrad, Irene Serrano-García, Cristina Fernández Pérez, Manuel Serrano-Ríos

**Affiliations:** 1 Spanish Biomedical Research Centre in Diabetes and Associated Metabolic Disorders (CIBERDEM), Madrid, Spain; 2 Instituto de Investigación Sanitaria del Hospital Clínico San Carlos (IdISSC), Madrid, Spain; 3 UGC de Medicina Preventiva, Hospital Clínico San Carlos, Madrid, Spain; 4 Facultad de Enfermería, Universidad Complutense de Madrid (UCM), Madrid, Spain; University of Cordoba, SPAIN

## Abstract

**Aim:**

To determine the association of body mass index (BMI), waist circumference (WC), waist to hip ratio (WHR), waist to height ratio (WHtr) and Body Shape Index (ABSI) with high cardiovascular risk (CVR), as well as to determine whether how strong are these relationships.

**Material and methods:**

A cross-sectional study was carried out in Spanish Caucasian adults. 3,456 subjects completed the study, 45.78% males, aged < 65 years and non-diabetic subjects. Anthropometric/biochemical variables were measured. We determined ABSI based on WC adjusted for height and weight. High CVR was defined as ≥ 20% according to the Framingham chart, ≥ 5% with the SCORE chart, and ≥ 7.5% with the ACC/AHA guide. Areas under the receiver operating characteristic curves (AUCs) were estimated for each anthropometric measure.

**Results:**

Most significant AUCs in males were: WHtr and ABSI for Framingham ≥ 20% and SCORE ≥ 5%. Also significant were WHtr, WC and ABSI for ACCA/AHA ≥ 7.5%. On the other hand, most significant AUCs in females were: WHtr and WC for Framingham ≥ 20%; and WHtr and WHR for SCORE ≥ 5%, WHtr, and WC for ACC/AHA guide ≥ 7.5%.

**Conclusions:**

Overall, the best anthropometric index identifying Spanish males and females who are at high risk for CV events is WHtr. ABSI was also found to be a good anthropometric index to predict high CVR in Spanish males according to FR, SCORE and ACC/AHA charts. For Spanish females, WC is a good anthropometric index according to FR and ACC/AHA guide, while WHR is better according to SCORE.

## Introduction

The distribution of adipose tissue has been related to cardiovascular risk factors and biochemical components of the metabolic syndrome (MS). The relationship between obesity and cardiovascular risk (CVR) is well established [1,2]. More specifically, there is evidence that metabolic risk correlates with the extent of visceral obesity, while subcutaneous fat is a source of protective adipokines [3].

Many studies have suggested variations in the ability to predict CVR morbidity and mortality in adults of body mass index (BMI), waist circumference (WC) and waist-to-hip ratio (WHR), which may result different according to different ethnics as well as age groups [4, 5]. WC and WHR reflect visceral fat, hence abdominal obesity. More recently waist-to-height-ratio (WHtr) has received attention worldwide for being strongly associated with several chronic diseases [5,6,7]. A new recently introduced anthropometric measure, named body shape index (ABSI), appears to be a substantial risk factor for premature mortality in the general population [8]. Moreover, ABSI could express the excess risk from high WC in a manner that is complementary to BMI and to other known risk factors [8]. In this context, the ABSI index has already demonstrated in other populations a significant correlation with mortality incidence [9] and risk [10].

Many clinical guidelines for cardiovascular disease (CVD) prevention contain risk estimation charts/calculators that have been developed to allow decision on the best management for patients at cardiovascular risk. Framingham Cardiovascular Risk Score [11], the European SCORE [12] and the most recently introduced pooled cohort studies equation from the American College of Cardiology/American Heart Association (ACC/AHA) [13] are widely and recently encouraged to be used as their theoretical flaws do not invalidate their utility as cardiovascular risk tools [14]. Moreover, each of those charts performs better according to each population characteristics: age, sex, ethnicity, etc… For this reason, and not only for clinical use but also for investigation, it is recommended to use more than one CV risk chart [15]. The most globally used score chart has been the Framingham Cardiovascular Risk equation, although for European populations the SCORE project developed a cardiovascular mortality risk chart for subjects up to 65 years old, differentiating countries at high vs. low cardiovascular risk such as Spain [12]. Framingham previous charts tend to overestimate the CV risk in Spanish population [16] but the most recent one designed for Primary Care [11] seems to be appropriate for Caucasian European Populations [17].

This study aims to determine the association, in a well characterized Spanish population, between body mass index (BMI), waist circumference (WC), waist to hip ratio (WHR), waist to height ratio (WHtr) and Body Shape Index (ABSI) with high cardiovascular risk (CVR) as evaluated by Framingham [11], SCORE [12] and ACC/AHA [13], as well as to determine whether the strength of association of BMI, WC, WHR, WHtr and (ABSI) indexes with the estimated CVR is, in fact, different.

## Material and methods

### Design, population

Details of recruitment and Study protocols of this population based survey were previously described [18–20]. In brief, there were 5,941 men and non-pregnant women aged 35–74 years, from a targeted population of 496,674 subjects from 21 small and middle-sized towns across Spain who were invited to participate. All subjects were sent a personalized post mail signed by the principal investigator and the authorities of the Regional Public Health Service, explaining the purpose of the study and requesting volunteering for participation. In case of no response, people were again contacted by telephone up to three times.

Two hundred and fifty-three subjects were excluded as they met one or more of the following exclusion criteria: type 1 diabetes mellitus, overt heart or hepatic failure, surgery in the previous year, weight changes (more than 5 kg gain or loss within the previous 6 months), and hospitalization for any reason at the time of participating in our study. 1,844 subjects were not recruited due to census errors or refusal to participate.

A total of 3,844 subjects (response rate 75.8%) completed the study, 1,754 males and 2,090 females. We used standard procedures adapted from the WHO MONICA protocol [21], approved by our Ethics Committee of Clinic Hospital San Carlos, Madrid. All participants were given written informed consent. For the purpose of this study, we excluded subjects over 65 years old and participants with diabetes (n = 388), therefore a total of 3,456 subjects were finally included. Trained interviewers obtained the data following a medical questionnaire that included age, sex, parity, menopausal status, family history of diabetes, treatment of diabetes, hypertension, and other relevant chronic diseases.

Anthropometric measurements included: Body Mass Index (BMI: kg/m2) and waist circumference (cm) (WC); whose cut-off points in the Spanish population (94.5/89.5 cm for males/females) have been previously reported [22]; and were considered to define abdominal obesity. Waist measurements were made with a non-stretchable fibre measuring tape while study participants were standing erect in a relaxed position with both feet together on a flat surface. WC was measured as the smallest horizontal girth between the costal margins and the iliac crests at minimal respiration. Hip circumference (HC) was measured at the level of the greater femoral trochanters. These measurements were used to compute WC divided by HC [waist-to-hip ratio (WHR)]. Waist to Height ratio (WHtr) is another proxy for central obesity that corrects the WC for the height of the individual [23–28].

The reliability of the anthropometric measurements was established by comparing values obtained by three different interviewers in a sample (n = 3,844) of individuals.

We defined A Body Shape Index (ABSI) according to the definition by Krakauer N.Y. et al. [8] based on WC adjusted for height and weight:

$$ABSI=\frac{WC}{{BMI}^{2/3}{Height}^{1/2}}$$

To estimate cardiovascular risk, we used the Framingham risk chart (FR) for primary care by D´Agostino et al. [11], the SCORE Cholesterol risk chart for low risk European regions [12] and the American College of Cardiology and the American Heart Association (ACC/AHA) guide [13]. The FR chart has a sensitivity and specificity of 48% and 85% in men and 58% and 83% in women, respectively. The SCORE risk chart for low risk regions has a 35% sensitivity and 88% specificity considering both sexes. The (ACC/AHA) guide has a great concordance according to the C-statistics test, where it has been described a range from 0.713 in African-American men to 0.818 in African-American women [13]. A CVR ≥ 20% according to the FR chart is associated with high risk of cardiovascular morbidity, a threshold CVR ≥ 5% with the SCORE chart indicates high risk of cardiovascular mortality [12], and finally, ACC/AHA guide classify subjects with a CVR ≥ 7.5% as high risk individuals for a first hard atherosclerotic cardiovascular disease event [13].

### Procedures and laboratory studies

After an overnight fasting period, 20 ml of blood were obtained from an antecubital vein without compression. Plasma glucose was determined duplicate by a glucose-oxidase method adapted to an Autoanalyzer (Hitachi 704, Boehringer Mannheim, Germany).

Total cholesterol, triglycerides and high-density lipoprotein cholesterol (HDL-C) were determined by enzymatic methods using commercial kits (Boehringer, Mannheim, Germany). Low density lipoprotein cholesterol (LDL-C) was calculated by the Friedewald formula [29]. A 75-g oral glucose tolerance test (OGTT) was performed and interpreted according to the revised 2003 criteria of the American Diabetes Association [30]. Diabetes mellitus was diagnosed when fasting plasma glucose was ≥ 7.0 mmol/l or 2-h post glucose ≥ 11.1 mmol/l. Subjects on antidiabetic medication were also considered as subjects with diabetes. In nondiabetic subjects, fasting plasma glucose of 5.6–6.9 mmol/l was indicative of impaired fasting glucose (IFG) and 2-h glucose of ≥ 7.8–11.0 mmol/l of impaired glucose tolerance (IGT). Serum insulin concentrations were determined by RIA (Human Insulin Specific RIA kit, Linco Research Inc., St Louis, MO, USA). This assay had a lower detection limit of 2 mU/ml with within and between assay coefficients of variation of 1% and 7.43%, respectively. Cross reactivity with proinsulin was under 0.2%. Insulin resistance (IR) was estimated by homeostasis model assessment of IR (HOMA-IR) using the following formula: fasting insulin (mU/ml) x fasting glucose (mmol/l)/22.5 [31]. In subjects without clinical or biological parameters of IR, the 90th percentile for the HOMA-IR was equal to or greater than 3.8, and this value was considered diagnostic of IR [32].

Study subjects were divided into three categories based on BMI: non-obese: BMI < 25 Kg/m2, overweight BMI 25–29.9 Kg/m2, and obese: BMI ≥ 30 Kg/m2. Women were considered pre-or post-menopause according to the NICE guidelines [33].

Finally, participants with diabetes mellitus were excluded because diabetes subjects are classified as high CVR subjects according to the SCORE chart. This would lead to an unrealistic comparison with the other risk charts where diabetes is considered “only” a major risk factor that adds significant risk but is not considered a “coronary equivalent” [11,12,13].

### Statistical analyses

Student t test or ANOVA were used to compare continuous variables expressed as means and standard deviation (SD), while categorical variables were compared using the Chi-squared test. The receiver operator characteristic curves (ROC) were conducted to evaluate the performance of the BMI, WC, WHR, WHtr, and ABSI anthropometric parameters in detecting Framingham risk ≥ 20%, SCORE risk ≥ 5% and ACC/AHA ≥ 7.5% by sex. We estimated differences in the area under the curve (AUC) with 95% confidence intervals (CI). Then, we compared in pairs the areas under the correlated receiver operating curves (AUCs) following the method by DeLong [34].

The level of significance was set at 0.05 for all analyses. All analyses were performed using Windows SPSS software version 15.0 (version 20.0; Inc., Chicago, IL, USA).

## Results

The clinical characteristics of the studied population, stratified by gender, are shown in Table 1. 45.78% (n = 1,582) of the subjects were males, and 54.22% (n = 1,874) were females.

**Table 1:** Characteristics of population by sex.

	Males N = 1,582	Females N = 1,874	p
	X (SD)	X (SD)	
Age (years)	48.57 (8.61)	48.83 (8.52)	0.378
Weight (Kg)	77.88 (11.78)	67.76 (12.60)	< 0.001
BMI (kg/m^2^)	27.42 (3.60)	27.59 (4.82)	0.247
WC (cm)	94.09 (9.66)	84.58 (10.83)	< 0.001
Hip (cm)	97.96 (8.53)	101.46 (10.19)	< 0.001
WHR	0.96 (0.07)	0.83 (0.07)	< 0.001
WHtr	0.56 (0.06)	0.54 (0.07)	< 0.001
ABSI	0.09 (0.01)	0.08 (0.01)	< 0.001
SBP (mm Hg)	125.66 (18.27)	124.83 (20.19)	0.211
DBP (mm Hg)	79.63 (11.22)	77.96 (11.44)	< 0.001
Fasting glucose (mg/dl)	91.97 (12.95)	88.09 (12.63)	0.099
Glucose 2 h (mg/dl)	97.41 (32.26)	100.62 (28.78)	0.007
Fasting Insulin (UI/ml)	12.61 (9.26)	12.72 (11.82)	0.779
Insulin 2 h (UI/ml)	31.91 (48.34)	41.90 (45.80)	0.389
Cholesterol (mg/dl)	222.24 (41.10)	217.03 (40.47)	< 0.001
Triglycerides (mg/dl)	131.03 (85.11)	95.08 (51.41)	< 0.001
HDL-Cholesterol (mg/dl)	47.90 (13.56)	57.14 (15.08)	< 0.001
LDL-Cholesterol (mg/dl)	148.13 (37.97)	140.95(36.87)	< 0.001
HOMA-IR	2.85 (2.23)	2.78 (2.66)	0.435
IR by HOMA-IR ≥ 3.8 (%)	19.5	17.1	0.078

ABSI: Body Shape Index, BMI: Body Mass Index, DBP: Diastolic Blood Pressure, HDL-Cholesterol: High Density Lipoproteins, HOMA-IR: Homeostasis Model Assessment, IR: Insulin Resistance, LDL-Cholesterol: Low Density Lipoproteins, SBP: Systolic Blood Pressure, WC: Waist Circumference, WHR: Waist Hip Ratio, Waist Height Ratio, X (SD): mean (standard deviation).

Women showed lower mean values of weight, WC, WHR, WHtr, ABSI, DBP, cholesterol, triglycerides. However, fasting HDL-C and glucose tolerance 2 hours level were higher in women than in men.

The overall prevalence of obesity in our population was 27.5% (23.7% in males and 30.2% in females); overweight 45.3% (53.1% in males and 38.6% in females) and normal weight 27.2% (23.7% in males and 31.2% in females).

**Fig 1:**
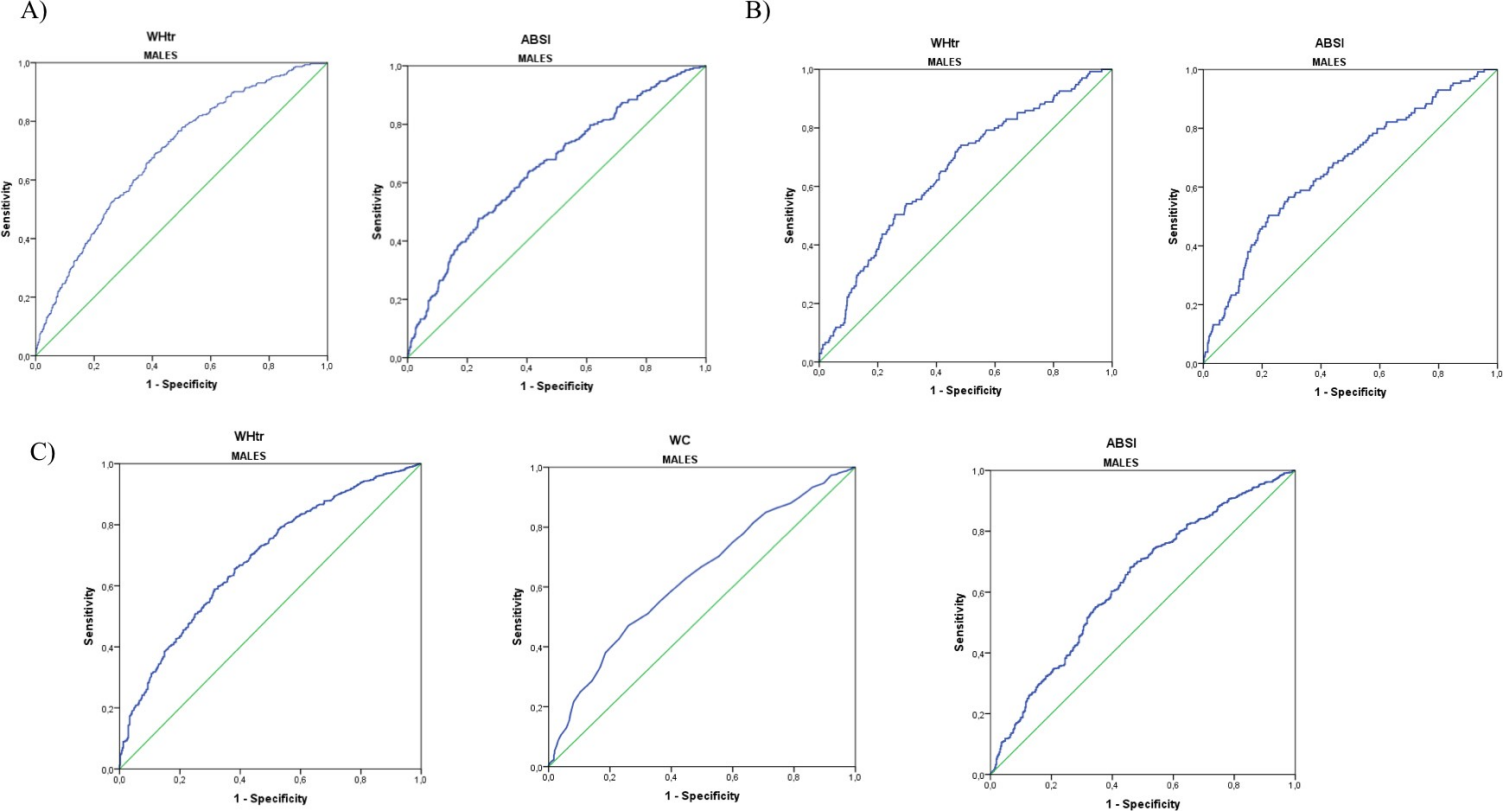
Most significant ROC curves of body fat anthropometric indexes in males according to high CVR. A) Estimated by Framingham (≥ 20%). B) Estimated by SCORE (≥ 5%). C) Estimated by ACC/AHA (≥ 7.5%).

**Fig 2:**
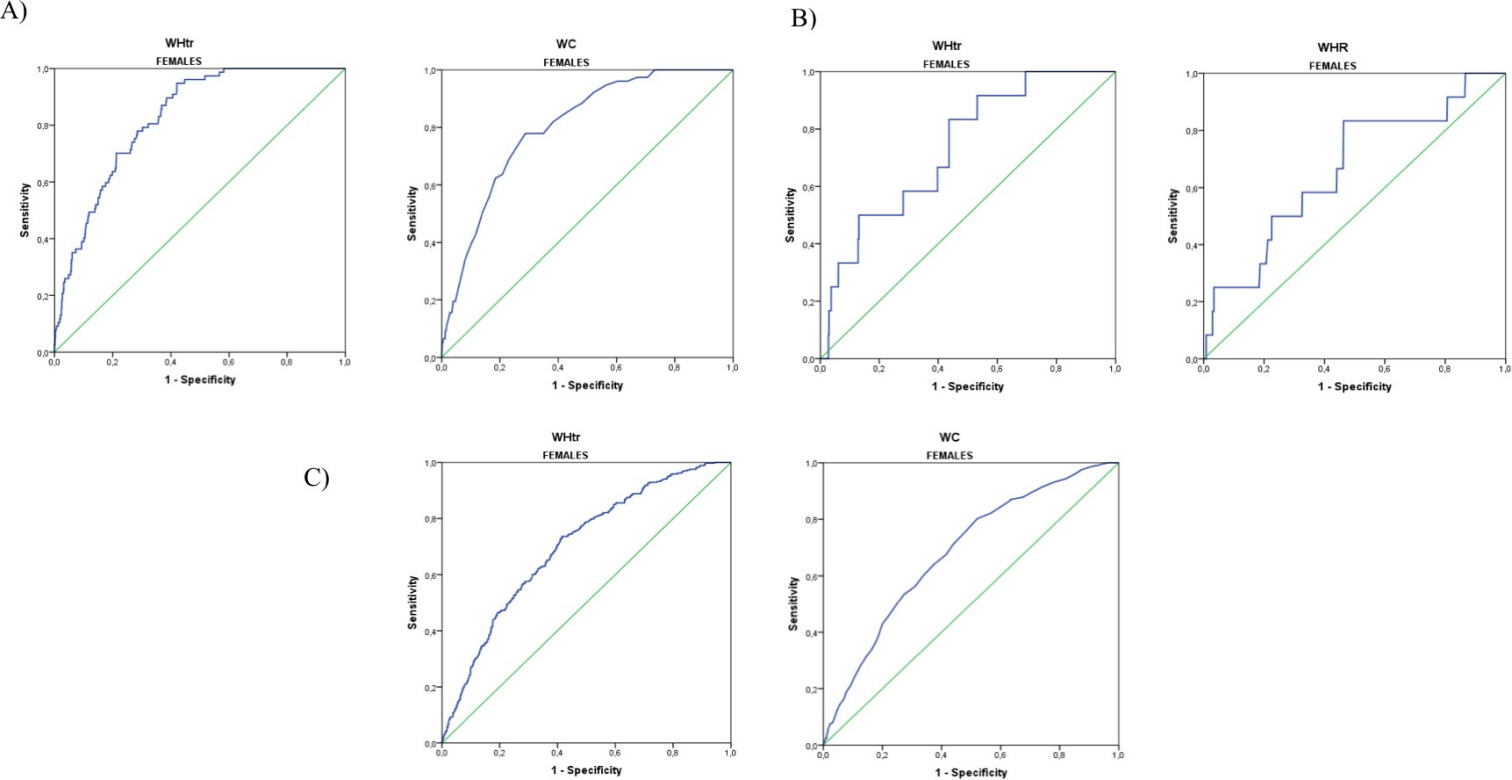
Most significant ROC curves of body fat anthropometric indexes in females according to high CVR. A) Estimated by Framingham (≥ 20%). B) Estimated by SCORE (≥ 5%). C) Estimated by ACC/AHA (≥ 7.5%).

In Table 2 we observed that the most significant AUCs in males were those that follow: 0.687 (95% CI = 0.655–0.720) for WHtr and 0.653 (95% CI = 0.617–0.689) for ABSI according to Framingham ≥ 20% (Fig 1A); 0.665 (95% CI = 0.605–0.701) for WHtr and 0.665 (95% CI = 0.615–0.715) for ABSI for SCORE ≥ 5% (Fig 1B & Table 3). Finally, for ACCA/AHA ≥ 7.5%, the most relevant AUCs were 0.687 (95% CI = 0.657–0.717) for WHtr, 0.632 (95% CI = 0.601–0.662) for WC and 0.631 (95% CI = 0.599–0.664) for ABSI (Fig 1C & Table 4). Regarding most significant AUCs in females, better correlations were obtained: 0.826 (95% CI = 0.790–0.862) for WHtr, 0.801 (95% CI = 0.758–0.844) for WC for Framingham ≥ 20% (Fig 2A & Table 2); and 0.733 (95% CI = 0.603–0.863) for WHtr, 0.662 (95% CI = 0.500–0.823) for WHR for SCORE ≥ 5%, (Fig 2B & Table 3). Finally, 0.699 (95% CI = 0.670–0.728) for WHtr and 0.683 (95% CI = 0.653–0.713) for WC for ACCA/AHA ≥ 7.5% (Fig 2C & Table 4).

**Table 2:** Areas under ROC curves of body fat anthropometric indexes according to high CV risk estimated with FRAMINGHAM (≥ 20%).

	MALES					FEMALES				
	AREA	CI 95%	Cut-off	Sensitivity	Specificity	AREA	CI 95%	Cut-off	Sensitivity	Specificity
**BMI**	0.591	0.555–0.626	26.80	0.681	0.481	0.788	0.744–0.832	28.79	0.779	0.663
**WC**	0.645	0.611–0.680	97.50	0.549	0.675	0.801	0.758–0.844	89.5	0.779	0.712
**WHR**	0.593	0.558–0.629	0.958	0.637	0.517	0.662	0.610–0.714	0.822	0.828	0.448
**WHtr**	0.687	0.655–0.720	0.564	0.691	0.588	0.826	0.790–0.862	0.546	0.948	0.579
**ABSI**	0.653	0.617–0.689	0.089	0.477	0.761	0.637	0.571–0.703	0.081	0.948	0.579

ABSI: Body Shape Index, BMI: Body Mass Index, WC: Waist Circumference, WHR: Waist to Hip Ratio, WHtr: Waist to Height Ratio.

CI: confidence interval.

**Table 3:** Areas under ROC curves of body fat anthropometric indexes according to high CV risk estimated with SCORE (≥ 5%).

	MALES					FEMALES				
	AREA	CI 95%	Cut-off	Sensitivity	Specificity	AREA	CI 95%	Cut-off	Sensitivity	Specificity
**BMI**	0.522	0.470–0.575	27,72	0.511	0.579	0.645	0.504–0.786	27.64	0.750	0.558
**WC**	0.579	0.528–0.631	101,50	0.325	0.810	0.653	0.477–0.828	94.50	0.583	0.826
**WHR**	0.550	0.499–0.601	0.958	0.600	0.500	0.662	0.500–0.823	0.836	0.833	0.536
**WHtr**	0.653	0.605–0.701	0.557	0.740	0.516	0.733	0.603–0.863	0.546	0.833	0.563
**ABSI**	0.665	0.615–0.715	0.090	0.503	0.777	0.625	0.428–0.822	0.083	0.545	0.760

ABSI: Body Shape Index, BMI: Body Mass Index, WC: Waist Circumference, WHR: Waist to Hip Ratio, WHtr: Waist to Height Ratio.

CI: confidence interval.

**Table 4:** Areas under ROC curves of body fat anthropometric indexes according to high CV risk estimated with ACC/AHA (≥ 7.5%).

	MALES					FEMALES				
	AREA	CI 95%	Cut-off	Sensitivity	Specificity	AREA	CI 95%	Cut-off	Sensitivity	Specificity
**BMI**	0.598	0.566–0.630	26.74	0.604	0.55	0.662	0.632–0.697	27.64	0.644	0.609
**WC**	0.632	0.601–0.662	96.50	0.469	0.741	0.683	0.653–0.713	81.50	0.802	0.478
**WHR**	0.601	0.573–0.638	0.957	0.577	0.601	0.621	0.590–0.652	0.810	0.780	0.412
**WHtr**	0.687	0.657–0.717	0.558	0.588	0.685	0.699	0.670–0.728	0.537	0.736	0.583
**ABSI**	0.631	0.599–0.664	0.089	0.477	0.761	0.609	0.576–0.643	0.081	0.625	0.626

ABSI: Body Shape Index, BMI: Body Mass Index, WC: Waist Circumference, WHR: Waist to Hip Ratio, WHtr: Waist to Height Ratio.

CI: confidence interval.

On the other hand, in Table 5 we show in a dichotomy way, according to the DeLong method, how the anthropometric parameters perform estimating CVRs. For males, the most significant anthropometric parameters related to CVR were ABSI and WHtr according to Framingham and SCORE charts, whereas for females the most significant anthropometric parameters were BMI, WC and WHtr according to Framingham. Regarding the ACC/AHA risk guide, the most significant parameters were WHtr in males and BMI, WC and WHtr in females. Furthermore, no superiority was found for any anthropometric parameter to detect higher CVR in females according to the SCORE chart. Finally, there were not pre-menopausal women at high cardiovascular risk according to the SCORE chart and only a 0.8% with the Framingham chart. In contrast, we found a 7.9% of pre-menopausal women at high CVR according to ACC/AHA. There were not significant differences of the anthropometric indexes AUCs, although BMI and WHtr showed a tendency to be different in both groups (p = 0.129 and 0.105 respectively).

**Table 5:** Comparison of anthropometric parameters by sex.

			p-value (DeLong Methods)	
			Females	Males
Framingham ≥ 20%		BMI—WC	0.6595	**0.0291**
		BMI—WHR	**0.0003**	0.9163
		BMI—ABSI	**0.0001**	**0.0160**
		BMI—WHtr	0.1858	**< 0.0001**
		WC—WHR	**0.0001**	**0.0029**
		WC—ABSI	**< 0.0001**	0.7713
		WC—WHtr	0.3899	0.0818
		WHR—ABSI	0.5610	**0.0214**
		WHR- WHtr	**< 0.0001**	**0.0001**
		ABSI—WHtr	**< 0.0001**	0.1637
**SCORE** ≥ 5%		BMI—WC	0.9446	0.1274
		BMI—WHR	0.8767	0.4502
		BMI- ABSI	0.8745	**< 0.0001**
		BMI—WHtr	0.3661	**0.0003**
		WC- WHR	0.9412	0.2287
		WC—ABSI	0.8382	**0.0192**
		WC—WHtr	0.4707	**0.0409**
		WHR—ABSI	0.7789	**0.0016**
		WHR—WHtr	0.4991	**0.0042**
		ABSI—WHtr	0.3703	0.7269
**ACC/AHA** ≥ 7,5%		BMI—WC	0.3315	0.1356
		BMI—WHR	0.0610	0.7328
		BMI—ABSI	**0.0214**	0.1479
		BMI—WHtr	0.0825	**< 0.0001**
		WC—WHR	**0.0043**	0.0869
		WC—ABSI	**0.0011**	0.9831
		WC—WHtr	0.4444	**0.0125**
		WHR—ABSI	0.6230	0.2695
		WHR—WHtr	**0.0002**	**0.0002**
		ABSI—WHtr	**< 0.0001**	**0.0133**

BMI: Body Mass Index, WC: Waist Circumference, WHR: Waist Hip Ratio, ABSI: Body Shape Index, WHtr: Waist Height Ratio. Statistical results have been highlighted in bold letters.

## Discussion

In this study, we analyse the association between several well recognized anthropometric parameters and its impact on CVR, evaluated by FR, SCORE and ACC/AHA charts, in adult males and females from the general population in Spain. For Spanish females, we have found that WC and WHtr seem to be the best anthropometric indexes to predict a higher CVR according to FR and ACC/AHA charts, while WHtr and WHR are the best according to the SCORE chart. In contrast, WHtr and ABSI are the best anthropometric parameters according to FR and SCORE charts for Spanish males, while WHtr, WC and ABSI demonstrate better correlation with CVR according to the ACC/AHA chart. It is noticeable that several CVR studies have been carried out in the Spanish population (IBERICA, DORICA, PREDIMED amongst others) [35–37], but the correlation of anthropometric parameters with the CVR has not been yet studied. Nevertheless, some international studies have contributed to study the relationship between anthropometric indexes and the prediction of cardiovascular events, metabolic variables and total mortality, in a diversity of populations. However, there is a paucity of information on the utility of WHtr and ABSI in assessing these risks among populations, especially in subjects with normal ranges of BMI and WC. Unfortunately, previous reports have shown inconsistent results for the utility of these obesity related indexes to assess cardiometabolic risks.

In a cross-sectional study among German adults, WC and WHtr were found to be better predictors of cardiovascular risk than BMI or WHR, although differences were small [38]. Another study [39] also showed that WC and WHtr were more strongly correlated with intra-abdominal visceral fat (as determined by Computed Tomography), indicating that these anthropometric parameters are best surrogate markers of the intraabdominal deleterious fat mass than BMI or WHR, probably explaining their relationship with higher CVR. Moreover, WHtr has shown not only to be a valid anthropometric index to diagnose obesity, but a good indicator to predict obesity and some non-communicable diseases in the elderly population [40].

On the other hand, some authors consider that WHtr index outperforms WC because it corrects the WC for the height of the individual, and they even question the relationship between WC and visceral fat mass [41, 42]. In this sense, other authors have found that WHtr increases the ability to predict cardiometabolic risk factors [28, 43, 44]. A similar result was found in a systematic review and meta-analysis carried out in 2012 with over 300,000 subjects that concluded that WHtr is the anthropometric parameter that best relates to cardiometabolic risk factors in both sexes and several ethnic and age groups, outperforming the predictive value of WC and BMI [43]. In short, these results are in accordance with our study, as WHtr has been the only anthropometric parameter systematically related to a high cardiovascular risk prediction according to the three risk charts in both sexes.

Otherwise, there are few studies comparing changes of body fat anthropometric parameters and their relationship with cardiovascular morbidity and mortality in premenopausal vs. menopausal women and we have not been able to demonstrate any differences at this point. WC was associated with higher global mortality in different postmenopausal ethnic women groups aged 50–79 years old [45]. On the other hand, WHR was suggested as a useful measurement predicting the regional obesity-associated metabolic abnormalities with their morbidity and mortality risk in pre-menopausal women [46]. These divergences could be in accordance with different degrees of visceral adiposity (VA), measured with computed tomography, that have been found in menopausal versus non-menopausal women [47]. To summarize, WHR seems to better correlate with the amount of VA, while results on WC are contradictory [47, 48].

There are also some other studies that have addressed the possibility of using composite indexes which include two or more anthropometric parameters, as for example the study by Millar et al. [49], that supported the use of WHtr together with BMI to improve body fat classification. In fact, cardiometabolic risk stratification using a composite index may provide a more accurate method for identifying subjects at high or low CVR. Even in a recent study by Bertoli S et al. [50], ABSI—as a surrogate marker for central obesity—was found to be a useful index for evaluating the independent contribution of WC, in addition to that of BMI. However, these high complexity indexes seem difficult to apply in daily clinical practice.

Finally, ABSI predicted mortality risk regardless age, sex, and weight in the NHANES study [9], Authors concluded that ABSI showed a stronger association with cardiovascular, cancer and global mortality, as compared with other reported anthropometric measures. However, the added mortality predictive value of ABSI was limited, and differences across ethnicities made authors conclude that more studies need to be carried out to draw conclusions.

### Study limitations

Causal inferences from our results are not possible because of the cross-sectional design. The initial sample size reduction could have conducted to a non-representative population study. Thus, we compared our cohorts (age, sex distribution and area frequencies of the included subjects) with the Spanish National Institute of Statistics Census (www.ine.es) for the same years and found that they were nearly identical. Other potential biases are: 1) we don’t have real events so cardiovascular risk has been estimated with charts, which nevertheless, are widely used for this purpose not only by clinicians but also in populations studies, as they allow to obtain a reliable approach to the future cardiovascular events [14, 17]; 2) our mean age study population was 48 years old, so our results might be, therefore, only applicable to middle aged populations; 3) we examined the anthropometric measures only once, at the moment of the study, for each subject. Therefore, no conclusions can be drawn regarding the changes in the anthropometric measures over time. However, the fortress of this study is its population-based design, with a wide number of Caucasian participants.

In summary, our findings suggest that overall, the best anthropometric index identifying Spanish males and females who are at high risk for CV events is WHtr. ABSI is also a good anthropometric index to predict high CVR in Spanish males according to FR, SCORE and ACC/AHA charts. For Spanish females, WC is a good anthropometric index according to FR and ACC/AHA guide, while WHR is better according to SCORE.

## Supporting information

S1 Table Dataset of the included Spanish population. The data file contains the following traits: Identification number (ID), Age, Gender, Smoke, Weight, Height, Waist circumference (WC), Waist to hip ratio (WHR), Waist to height ratio (WHtR), Cholesterol, HDL cholesterol, Systolic blood pressure (SBP) and Menopause. When using this data, please cite the original publication.
(CSV)

## Abbreviations

ABSI: Body Shape Index
ACC/AHA: American College of Cardiology and American Heart Association
AUC: receiver operating characteristic curve
BMI: body mass index
CVR: cardiovascular risk
FR: Framingham risk chart
WC: waist circumference
WHtr: waist to height ratio
WHR: waist to hip ratio
